# Baicalein has protective effects on the 17β-estradiol-induced transformation of breast epithelial cells

**DOI:** 10.18632/oncotarget.14433

**Published:** 2017-01-02

**Authors:** Yan Chen, Jing Wang, Duan-Yang Hong, Lin Chen, Yan-Yan Zhang, Yi-Ni Xu, Di Pan, Ling-Yun Fu, Ling Tao, Hong Luo, Xiang-Chun Shen

**Affiliations:** ^1^ The High Educational Key Laboratory of Guizhou Province for Natural Medicinal Pharmacology and Druggability, Guizhou Medical University, Huaxi university town, Guian new district 550025, Guizhou, People’s Republic of China; ^2^ Department of Pharmacology of Chinese Material Medica, Guizhou Medical University, Huaxi university town, Guian new district 550025, Guizhou, People’s Republic of China

**Keywords:** baicalein, breast cancer, chemoprevention, 17β-estradiol, estrogen receptor

## Abstract

Epidemiologic and systematic studies have indicated that flavonoid consumption is associated with a lower incidence of breast cancer. Baicalein is the primary flavonoid derived from the roots of *Scutellaria baicalensis* Georgi. In the current study, the long-term exposure of breast epithelial cells to 17β-estradiol (E2) was used to investigate the chemopreventive potential of baicalein on neoplastic transformation. The results demonstrated that baicalein significantly inhibited E2-induced cell growth, motility, and invasiveness, and suppressed E2-induced misshapen acini formation in 3D cultures. Furthermore, it inhibited the ability of E2-induced cells to form clones in agarose and tumors in NOD/SCID immunodeficient mice. Docking studies using Sybyl-X 1.2 software showed that baicalein could bind to both estrogen receptor-α (ERa) and G-protein coupled estrogen receptor 30 (GPR30), which are two critical E2-mediated pathways. Baicalein prevented the E2-induced ERa-mediated activation of nuclear transcriptional signaling by interfering with the trafficking of ERa into the nucleus and subsequent binding to estrogen response elements, thereby decreasing the mRNA levels of ERa target genes. It also inhibited E2-induced GPR30-mediated signal transduction, as well as the transcription of GPR30-regulated genes. Therefore, these results suggest that baicalein is a potential drug for reducing the risk of estrogen-dependent breast cancer.

## INTRODUCTION

Breast cancer is the most commonly diagnosed cancer and the second leading cause of cancer-related deaths in women; it accounts for 29% of all new cancer diagnoses in women globally [1]. Although breast cancer prevention has received less attention than treatment, the primary prevention strategies including maintaining a healthy body weight, regular physical activity, moderating the alcohol intake, and chemoprevention have been identified and applied [2]. A lifetime of exposure to elevated levels of endogenous or exogenous estrogen is a key risk factor in the occurrence of breast cancer [3]. The long-term exposure to estrogen induces transformation phenotypes and genomic changes in breast epithelial cells including high invasiveness, loss of the ability to form organized and growth-arrested spheroid acini, and ultimately a modified glandular architecture and biological characteristics in developing primary breast cancers [4, 5]. Two different but complementary estrogen pathways have been identified. Oxidative metabolites of estrogen and estrogen receptor-mediated genomic and nongenomic signal transduction contribute to the carcinogenicity of estrogen, and thus could initiate, promote, or progress breast cancer [6]. Therefore, blocking the function of estrogen receptors and reducing its synthesis or metabolism may have implications for the development of novel preventive and therapeutic interventions for breast cancer.

The biological actions of estrogens are mainly mediated by binding to and activating the classical estrogen receptors (ERs), ERα and ERβ, which belong to the nuclear receptor superfamily [7]. In the classical pathway, ERα acts as a ligand-activated transcription factor, undergoes conformational changes, unbinds from heat shock proteins, and forms homo- or heterodimers. The dimer then translocates to the cell nucleus where it binds to the estrogen response elements (EREs), either directly or indirectly via transcription factors, and regulates the expression of estrogen-responsive genes [8]. ERα is expressed in 50–80% of breast tumors and its expression is a good indicator for anti-estrogen endocrine therapy [9]. However, some ERα-positive patients do not respond to endocrine therapy, and some ERα-negative breast cancer cells remain estrogen-responsive. This suggests the existence of an alternative receptor for estrogen.

G-protein coupled estrogen receptor 30 (GPR30, formerly known as GPER) is another estrogen-responsive receptor that functions independently of ERα. It activates both rapid non-genomic signaling and transcriptional events. GPR30 mediates estrogen responses via a mechanism involving activating the non-receptor tyrosine kinase SRC by the Gβγ subunit, which stimulates matrix metalloproteinases (MMPs) to release the active mature form of heparin-binding epidermal growth factor (HB-EGF) from membrane-bound pro-HB-EGF. Subsequently, the HB-EGF transactivates the epidermal growth factor receptor (EGFR) and the downstream extracellular signal-regulated kinase (ERK) and serine/threonine kinase AKT though the serine-threonine kinase RAF and phosphatidylinositol 3 kinase (PI3K), respectively. Both ERK and AKT ultimately regulate the expression of target genes including *FOS*, *JUN*, *EGR1*, *CTGF*, and *ATF3* [10].

Epidemiologic studies and systematic analyses have suggested that flavonoids exhibit promising results in chemoprevention and therapy for breast cancer [11]. Some studies have attributed the striking differences in the incidence of breast cancer between Asian and western women to dietary flavonoids intake [12]. Nevertheless, the association between dietary flavonoid intake and the risk of breast cancer remains controversial. However, Chang et al. demonstrated that the intake of flavonols and flavones, but not other flavonoid subclasses or total flavonoids, is associated with a decreased risk of breast cancer, especially among post-menopausal women [13].

**Figure 1:**
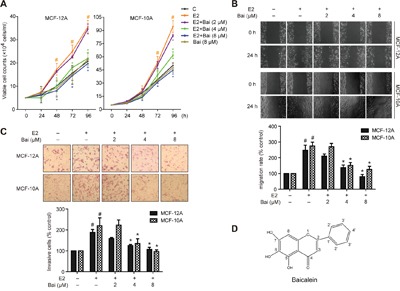
Baicalein prevents E2-induced cell growth, migration, and invasion in mammary epithelial cells Cells were treated with E2 or E2 plus baicalein (Bai) for 5 weeks and were then used in the following experiments. **A**. Cell growth was measured using trypan blue exclusion assay. The cell growth curve represents the growth of cells in the different treatment groups over 4 days. **B**. Cell migration was measured using wound healing assay. Confluent monolayers were scratched and incubated in serum-free culture medium; images were captured at 0 and 24 h after wounding (magnification, ×100). The level of cell migration into the wound scratch was quantified as migration rate by comparing with the control (as 100%). **C**. Cell invasion was investigated using the Matrigel-coated transwell model. Invasive cells that passed through the membrane were evaluated using H&E staining (magnification, ×200). The results are expressed as invasive cells with respect to the control (as 100%). **D**. Chemical structure of baicalein. The images are representative of three independent experiments. Data are shown as means ± SEM (n = 3). *P < 0.05 vs. E2, ^#^P < 0.05 vs. control.

Baicalein is the primary flavone derived from Radix Scutellariae, the traditional Chinese medicinal herb Huang Qin; it bears the three-ring structure of the flavone backbone with phenolic hydroxyl groups at the 5′, 6′, and 7′ positions (Figure 1D). It possesses a remarkable spectrum of pharmacological activities and extensive antitumor properties. It exerts potential effects on the treatment of breast cancer via complicated mechanisms including inducing cell cycle arrest and apoptosis and inhibiting cell proliferation, migration, invasion, and the epithelial-mesenchymal transition (EMT) [14]. It was shown that flavonoids contain a polyphenolic ring that is structurally similar to the steroid nucleus of 17β-estradiol (E2), and they may exhibit estrogenic or anti-estrogenic activity [15]. Previous studies found that baicalein inhibits E2-induced ER transactivation in MCF-7 cells and displaces >85% of estradiol binding in mouse uterine cytosol [16, 17]. Furthermore, we recently demonstrated that baicalein suppresses the E2-induced migration, adhesion, and invasion of breast cancer cells by disrupting GPR30 signaling in MCF-7 and SK-BR-3 breast cancer cells [18]. Taken together, these studies suggest that baicalein may exert anti-estrogenic activity and interfere with E2-induced ERα and GPR30 signaling transduction.

The present study investigated the ability of baicalein to prevent the E2 long-term exposure-induced transformation of non-tumorigenic MCF-12A and MCF-10A mammary epithelial cells using *in vitro* and *in vivo* models. Furthermore, the ability of baicalein to inhibit E2-induced ERα and GPR30 signaling activation in these cells was discussed. The chemopreventive effects of baicalein on E2-induced normal epithelial cell transformation and its inhibitory effects on the two estrogen receptors may provide a novel, promising approach toward breast cancer prevention.

## RESULTS

### Baicalein inhibits E2-enhanced cell growth, migration, and invasion in mammary epithelial cells

Since long-term exposure of E2 leads to the neoplastic transformation of human breast epithelial cells, the current study assessed the protective effects of baicalein by continuously treating non-tumorigenic MCF-12A and MCF-10A cells with E2 (20 nM) with or without baicalein (2, 4, or 8 μM) for 5 weeks. These concentrations of baicalein used in the experiments did not cause toxicity in the two cells (data not shown). Then, cell growth was examined on four consecutive days using trypan blue dye exclusion assay. Both cell lines grew faster than controls after treatment with E2. But baicalein inhibited E2-promoted cell growth (Figure 1A). Additionally, treatment with baicalein at 8 μM alone did not cause significant difference of the cell growth compared with control. Specifically, treatment with E2 for 5 weeks also elicited a significantly higher migration capacity in MCF-10A and MCF-12A cells; however, baicalein treatment caused a clear reduction in the width of the wound healing compared with E2 (Figure 1B). Next, transwell chamber assays were used to test the ability of MCF-12A and MCF-10A cells to invade through Matrigel-coated filters after treatment with E2 or E2 plus baicalein. The results showed that the invasive capability of both cell lines increased markedly when exposed to E2 for 5 weeks. However, baicalein significantly reduced the number of cells that crossed the Matrigel-coated filters compared with E2 (Figure 1C).

### Baicalein protects E2-disrupted acini grown in 3D cultures

Human mammary epithelial cells cultured in reconstituted basement membrane (Matrigel) form differentiated acini that resemble the acinar structures of mammary lobules [19]. This 3D epithelial culture model provides the appropriate structural and functional context for studying the features of the breast epithelium *in vivo*, as well as the features of malignant transformation and breast cancer [4]. Previous studies showed that the development process includes the initial proliferation of a single cell, the deposition of the basement membrane, and differentiation into two populations during the early stages of morphogenesis (a well-polarized outer layer and a poorly polarized inner layer). Then, the non-polarized inner cells began to die by apoptosis, which coincides with the formation of a hollow lumen [20].

**Figure 2:**
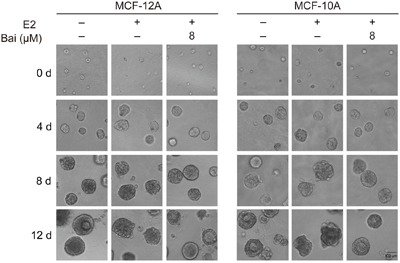
Analysis of the formation of acini in 3D cultures over time Cells were treated with E2 or E2 plus baicalein (8 μM) for 5 weeks, and were then allowed to grow in Matrigel for 12 days. Images were captured at 0, 4, 8, and 12 days after seeding under a microscope at 400× magnification. The photographs are representative of three independent experiments.

Therefore, this model was used to further analyze the effects of baicalein on the E2-induced transformation of mammary epithelial cells. After the indicated treatment for 5 weeks, cells were seeded in Matrigel and observed at 0, 4, 8, and 12 days to follow the development of acinar structures in a basement membrane gel. Cells in all groups were seeded as single cells (Figure 2). On the fourth day after plating, the acini remained small, and the two-layer organization was not observed; there were no clear differences between groups. By 8 days in culture, the acini were larger in size. Those in the control groups were rounded and well-organized, and had differentiated into two populations. However, the acini derived from E2-treated cells differed from controls since they exhibited an irregular spheroid shape and the two-layer organization was unclear. In contrast, the shape of acini was rounded and the two layers were visible in cells co-cultured with baicalein and E2; the acini disruption caused by E2 was reversed in the presence of baicalein. Finally, normal MCF-12A and MCF-10A cells that had been cultured for 12 days formed organized spheroid acini with hollow lumens. Conversely, the acinar structures formed by E2-treated cells were severely misshapen: they formed a disorganized acinar morphology with a filled lumen, and some formed complex multi-acinar structures. The distorted morphology of the acinar structures suggested excessive proliferation and indicated that the cells had escaped the normal phenotypic proliferative arrest that is characteristic of acinar development. Cells treated with the combination of E2 and baicalein exhibited normal structural features, with a spheroid shape and luminal clearing, yielding structures that resembled the control acini in many ways.

### Baicalein impedes E2-induced tumorigenesis *in vitro* and *in vivo*

**Figure 3:**
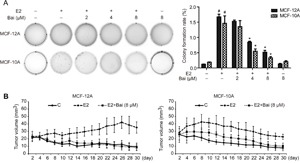
The effects of baicalein on E2-induced tumorigenesis in vitro and in vivo **A**. Cell colonies were grown in soft agar and cultured for 21 days after treatment with E2 or E2 plus baicalein for 5 weeks. Images of representative colonies were captured using a ChemiDoc XRS+ system; original magnification, 1×. Statistical analysis shows the colony formation rate relative to the total number of cells. Data are shown as means ± SEM (n = 3). *P < 0.05 vs. E2, ^#^P < 0.05 vs. control. **B**. Cells (2 × 10^6^ in 50 μl Matrigel) were implanted into the mammary fat pads of NOD/SCID mice after the indicated treatment for 10 weeks (n = 6 per group). The tumor volume was calculated by caliper measurements every other day from the time of detection for 30 days. The results are presented as mean tumor volume ± SEM (n = 6) and are representative of three independent experiments.

After culture with E2 or E2 plus baicalein for 5 weeks, MCF-12A and MCF-10A cells were transferred to soft agar to assess anchorage-independent growth. Both cell lines treated with E2 showed a significant increase in colony formation in soft agar (Figure 3A), which is a typical feature of cancer cells. However, the colony number and colony size were significantly reduced in MCF-12A and MCF-10A cells treated with E2 plus baicalein compared with E2 alone. Moreover, treatment with baicalein at 8 μM alone did not change the clone formation ability of the two cells compared with control. Based on these *in vitro* results, we next tested the inhibitory effects of baicalein on E2-induced tumorigenic ability *in vivo*. MCF-12A or MCF-10A cells were treated with E2 or E2 plus baicalein for 10 weeks, mixed with Matrigel at a volumetric ratio of 1:1, and injected into the mammary fat pads of female NOD/SCID mice (50 μl containing 2 × 10^6^ cells). The growth of these cells *in vivo* was monitored every other day for 30 days. As shown in Figure 3B, the MCF-12A and MCF-10A tumor volume in the control groups reduced gradually, and no colonies were observed after 30 days. E2-treated MCF-12A or MCF-10A cells formed colonies in the mammary fat pads after 30 days. However, the cells treated with E2 and baicalein (8 μM) were unable to form colonies or grow in mammary fat pads.

### Molecular modeling and docking of E2 and baicalein to ERα and GPR30

**Figure 4:**
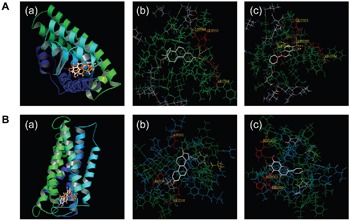
The binding of E2 and baicalein with ERα and GPR30 **A**. Docking analyses for the binding of E2 and baicalein with ERα. (a) The three-dimensional model of E2 (orange) and baicalein (white) to ERα. (b) The binding of E2 to ERα is shown; hydrogen bonds with Glu 353, Leu 349, and Arg 394 sites were formed. (c) The binding of baicalein to ERα is shown, and hydrogen bonds with Glu 353, Leu 387, Leu346, and Arg 394 sites were formed. **B**. Docking analyses for the binding of E2 and baicalein to GPR30. (a) A three-dimensional model of the binding of E2 (orange) and baicalein (white) to GPR30. (b) The binding of E2 to GPR30 is shown, and hydrogen bonds with His 197, Asp 202, and Glu 218 sites were formed. (c) The binding of baicalein to GPR30 is shown, and hydrogen bonds with His 282, Asp 202, and His 200 sites were formed.

To explore the possibility that baicalein blocks ERα and GPR30 signaling to disrupt the effects of E2, SWISS-MODEL and Sybyl-X 1.2 were used to evaluate the potential affinity of E2 and baicalein for the Ligand binding domains (LBDs) of ERα and GPR30 (Figure 4). When E2 was docked in the LBD of ERα (Figure 4A(a) and 4A(b)), three hydrogen bonds formed with Glu 353, Leu 349, and Arg 394 sites. Baicalein also formed hydrogen bonds in the ERα LBD with Glu 353, Leu 387, Leu 346, and Arg 394 sites (Figure 4A(a) and 4A(c)). In the GPR30 model, E2 bound to the GPR30 LBD by forming hydrogen bonds with His 197, Asp 202, and Glu 218 sites (Figure 4B(a) and 4B(b)). And baicalein was formed hydrogen bonds with His 282, Asp 202, and His 200 in the LBD of GPR30 (Figure 4B(a) and 4(c)).

### Baicalein inhibits E2-induced ERα nuclear translocation and transcriptional activity

**Figure 5:**
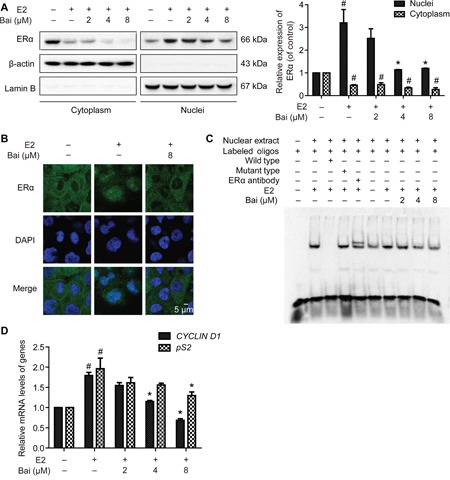
The effects of baicalein on E2-induced ERα nuclear transcriptional activation After pre-incubation in serum-free medium for 24 h, the cells were treated with E2 or E2 plus baicalein under the same condition for 24 h. **A**. The localization of ERα in cytoplasmic and nuclear fractions was analyzed by western blotting. The cytoplasmic and nuclear levels of ERα were normalized to β-actin and lamin B, respectively. **B**. The subcellular localization of ERα was confirmed by confocal microscopy. Images of ERα (green fluorescence) and cell nuclei stained with DAPI (blue fluorescence) are shown (×1000). Scale bar = 5 μm. **C**. EMSA was used to detect the binding of ERα to estrogen response elements (EREs). The composition of the DNA-binding complex was investigated using anti-ERα antibodies in supershift experiments. The concentration of the unlabeled wild-type or mutant probes (as competitor oligonucleotides) was 100-times greater than that of the labeled wild-type probe. **D**. The effects of baicalein on E2-induced ERα-regulated gene expression were analyzed using RT-PCR. The results obtained from experiments were normalized to *GAPDH* expression and are shown as the fold-change compared with control cells. Data are shown as means ± SEM (n = 3). *P < 0.05 vs. E2, ^#^P < 0.05 vs. control.

After pre-incubation in serum-free medium for 24 h and followed by treatment with E2 or E2 plus baicalein under the same condition for 24 h, the intracellular localization of ERα was analyzed in subcellular protein fractions from MCF-12A cells. ERα was observed in the cytoplasmic and nuclear compartments in control. When treatment with E2, the nuclear abundance of ERα increased significantly, whereas the amount of cytoplasmic ERα decreased significantly (Figure 5A). In contrast, baicalein significantly decreased the E2-induced nuclear translocation of ERα. Baicalein also reduced the amount of ERα in the cytoplasm. The cytoplasmic protein β-actin and the nuclear protein lamin B were used as loading controls and to confirm lysate purity. Next, immunostaining and confocal microscopy were used to confirm the effects of baicalein on the subcellular localization of ERα in MCF-12A cells that had been treated with E2. Under serum-free conditions, ERα was present mainly in the cytoplasm. However, treatment with E2 resulted in the obvious nuclear localization of ERα; the nuclear localization of ERα was inhibited in the presence of baicalein (Figure 5B).

The effects of estrogen on gene expression in mammary cells are mediated by the interaction between the ERα and EREs in target DNA. To investigate whether baicalein influences E2-induced ERα-ERE complex formation, EMSAs were performed with nuclear extracts isolated from cells and oligonucleotides containing the consensus EREs (“GGTCA” and “TGACC”) [21]. When the probes were incubated with nuclear extracts, significant ERα-EREs complex formation was observed in extracts from E2-treated cells. However, the intensity of the band decreased with concurrent baicalein treatment (Figure 5C). Unlabeled wild-type ERE oligonucleotide (cold probe), but not a mutant ERE oligonucleotide, competed for binding in the ERα complex. Furthermore, a specific anti-ERα antibody was used to form a supershifted complex, confirming that the complex contained ERα. Finally, we investigated whether baicalein modulates the expression of ERα-regulated estrogen-responsive genes, including *pS2* and *CYCLIN D1*, [22–24]. As shown in Figure 5D, the E2-induced upregulation of *pS2* and *CYCLIN D1* was significantly inhibited in the presence of baicalein.

### Baicalein prevents E2-induced GPR30-mediated signal transduction and gene expression

**Figure 6:**
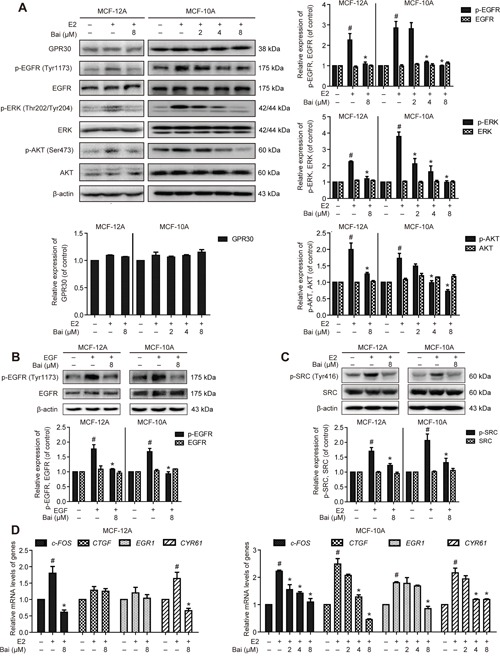
The effects of baicalein on E2-induced GPR30-mediated signal transduction and gene expression **A**. The cells were pre-incubated with serum-free medium for 24 h, and were then treated with E2 or E2 plus baicalein under the same condition for 1 h. Western blotting was used to examine the expression of GPR30 and the total and phosphorylated protein levels of EGFR, ERK1/2, and AKT in MCF-12A and MCF-10A cells. β-actin was used as the loading control. **B**. The cells were pre-incubated with serum-free medium for 24 h, and then treated with 100 ng/ml EGF for 10 minutes in the presence or absence of baicalein, the total and phosphorylated protein levels of EGFR were detected. **C**. With the treatment condition as (A), the total and phosphorylated protein levels of SRC were analyzed. **D**. With the treatment condition as (A), the mRNA expression of *c-FOS*, *CTGF*, *EGR1*, and *CYR61* was detected using RT-PCR. The results obtained from experiments were normalized to *GAPDH* levels and are presented as the fold-change compared with control cells. Data are shown as means ± SEM (n = 3). *P < 0.05 vs. E2, ^#^P < 0.05 vs. control.

The cells were pre-incubated with serum-free medium for 24 h, and were then treated with E2 or E2 plus baicalein under the same condition for 1 h. GPR30 expression was unchanged by exposure to E2 with or without baicalein in both MCF-12A and MCF-10A cells (Figure 5A). This suggests that E2 and baicalein do not directly influence GPR30 expression. Previous studies suggested that GPR30 activation triggers the MMP-dependent transactivation of EGFR, as well as the activation of ERK and AKT [25]. GPR30 also triggers transcriptional responses to upregulate the expression of its target genes including *c-FOS*, *EGR1*, *CYR61*, and *CTGF* [26]. Thus, we next evaluated whether baicalein inhibits GPR30-mediated signal transduction and gene expression. E2 treatment significantly increased EGFR phosphorylation at Tyr 1173; however, EGFR phosphorylation was decreased in the presence of baicalein (Figure 6A). Similarly, the E2-induced phosphorylation of ERK1/2 and AKT at Thr 202/Tyr 204 and Ser 473, respectively, was suppressed by co-treatment with baicalein. Meanwhile, the EGFR activation induced by EGF in MCF-12A and MCF-10A cells was prevented in the presence of baicalein (Figure 6B). The non-receptor tyrosine kinase SRC is required for the cascades of GPR30 signal transduction [27]. The phosphorylation at Tyr 416 in the activation loop of the SRC kinase domain indicates the increased enzyme activity [28]. Accordingly, the phosphorylation of SRC at Tyr 416 triggered by E2 was also inhibited by baicalein (Figure 6C), hence confirming its inhibitory activity on GPR30-mediated signaling. Furthermore, the E2-induced upregulation of *c-FOS*, *CTGF*, *CYR61*, and *EGR1* in MCF-10A cells and *c-FOS* and *CYR61* in MCF-12A cells was also suppressed by the presence of baicalein (Figure 6D).

## DISCUSSION

Advances in the diagnosis, surgical treatment, radiotherapy, and chemotherapy have reduced the breast cancer-associated mortality in the past 20 years [29]. However, the incidence of breast cancer has increased steadily, even in many Asian countries where the incidence of breast cancer is traditionally low [2]. It is estimated that more than half of all breast cancers could be prevented by healthy behavior and chemoprevention [2]. Thus, reducing the incidence of breast cancer requires the development of more effective methods for prevention.

Human breast cancer development is often characterized by an early stage involving the transformation of normal breast epithelial cells into mutant cells. These cells then become transformed into benign tumor cells, which subsequently progress into malignant cells. Under prolonged estrogen stimulation, breast epithelial cells have been confirmed to go transformation and tumorigenesis [30]. The current experimental also revealed that the long-term treatment of MCF-12A and MCF-10A cells with E2 resulted in an accelerated growth ability, increased motility and invasiveness, and the formation of misshapen acini with filled lumen in Matrigel, which together resemble the events that occur during the early stages of breast cancer in humans [31]. Moreover, E2-treated cells were able to form colonies in agarose and tumors when injected into NOD/SCID immunodeficient mice with Matrigel, both of which are potentially indicative of a cancerous phenotype. Nevertheless, when the cells were co-treated with E2 and baicalein, the cells exhibited decreased growth, a reduced migration and invasion ability compared with E2-treated cells, and formed 3D duct-like structures similar to control cells. The cells also did not have the potential to form colonies and tumors. These results suggest that baicalein can interfere with the E2-induced neoplastic transformation of the breast epithelium. Therefore, baicalein could be a potential chemopreventive agent against the transformation of a normal cell into a tumor cell during breast cancer initiation.

The association between breast cancer and ERs is very well known. Altered ERs signaling has been implicated in abnormal cell proliferation, as well as the carcinogenesis and progression of breast cancer [32]. In particular, ERα stimulates cell proliferation and the mutations arising from the replicative errors that occur during pre-mitotic DNA synthesis in estrogen-mediated carcinogenesis [33]. The selective ERα modulator tamoxifen is used to reduce the incidence of both invasive and non-invasive breast cancer in women at high risk for the disease [34]. A previous study demonstrated that baicalein suppresses E2-induced ERα transactivation in MCF-7 cells [16]. The current study demonstrated that baicalein interferes with the nuclear transcription function of ERα by impeding the E2-induced nuclear localization of ERα and subsequent binding to EREs containing “GGTCA” and “TGACC”. Baicalein also suppressed the E2-enhanced transcription of *pS2* and *CYCLIN D1*, which are two well-established ERα target genes [35] [36]. It was indicated that baicalein interferes with E2-induced ERα signaling activation.

Accumulating evidence has suggested that various anti-estrogens such as tamoxifen, raloxifen, and fulvestrant (ICI 182.780) function as GPR30 agonists, similar to E2, which in turn leads to cancer cell proliferation, migration, and invasion [37]. Recent literature has revealed that GPR30 is involved in the development of tamoxifen resistance [38] and that it correlates with clinical and pathological biomarkers of poor outcome in breast cancer [39]. Notably, GPR30 contributes E2-induced proliferation in breast epithelial cells and both normal and tumorigenic human breast tissue explants [27]. GPR30 is also overexpressed in invasive breast cancer, and its expression is positively correlated with the development of distant metastases [39, 40]. Additionally, GPR30 knockout MMTV-PyMT mice exhibit less aggressive tumors and fewer metastases [27]. Therefore, GPR30 signaling seems to be an additional mechanism that maintains the responsiveness of breast cancer cells to E2, thereby facilitating tumor progression and antiestrogen resistance. Targeting both ERα and GPR30 may be a rational pharmacological strategy for the treatment of breast cancer.

The current results demonstrated that baicalein significantly suppressed the E2-induced phosphorylation of SRC at Tyr 416, EGFR at Tyr 1173, ERK1/2 at Thr 202/Tyr 204, and Akt at Ser 473 in both MCF-10A and MCF-12A cells, a mechanism that was described previously for the activation of GPR30 signaling [41, 42]. Meanwhile, baicalein inhibits E2-induced GPR30-mediated target gene expression by preventing E2-enhanced *c-FOS*, *CTGF*, *EGR1*, and *CYR61* mRNA levels [26]. Taken together, these results suggest that baicalein exerts dual inhibitory effects on E2-mediated ERα and GPR30 signaling. In the present study, molecular modeling was used to assess whether baicalein binds to ERα and GPR30. However, it remains unclear whether baicalein could act as an antagonist of both receptors and the detailed mechanisms by which baicalein influences the downstream signaling need to be addressed. Furthermore, the possible existence of cross-talk between ERα and GPR30 in E2-induced carcinogenesis and whether it could be influenced by baicalein should also be investigated.

Although flavonoids have been studied for ∼60 years, the cellular mechanisms behind their biological activity remain largely unknown. The anticancer activities of baicalein have been attributed to a variety of mechanisms that are important in breast cancer, including inhibiting 12-LOX activity [43], upregulating DDIT4 expression, inhibiting mTOR [44], and suppressing the aromatase (CYP19) activity and thus decreasing estrogen biosynthesis [45]. Several studies have revealed that baicalein has good safety profile in preclinical and clinical studies. For example, it is not mutagenic, does not cause potentially detrimental genomic instability, and does not appear to be toxic in mice [44, 46]. It also protects against benzo[*a*]pyreneand aflatoxin B1-induced genotoxicity [47]. Furthermore, the safety of orally administered baicalein in healthy Chinese subjects was confirmed in a Phase I, randomized, double-blind, single-dose clinical trial [48]. It was safe and well-tolerated at single oral doses of 100–2800 mg and no serious adverse events were reported. Thus, these findings raise the possibility that baicalein is an attractive candidate anti-cancer drug and that further studies are warranted toward its clinical development.

**Figure 7:**
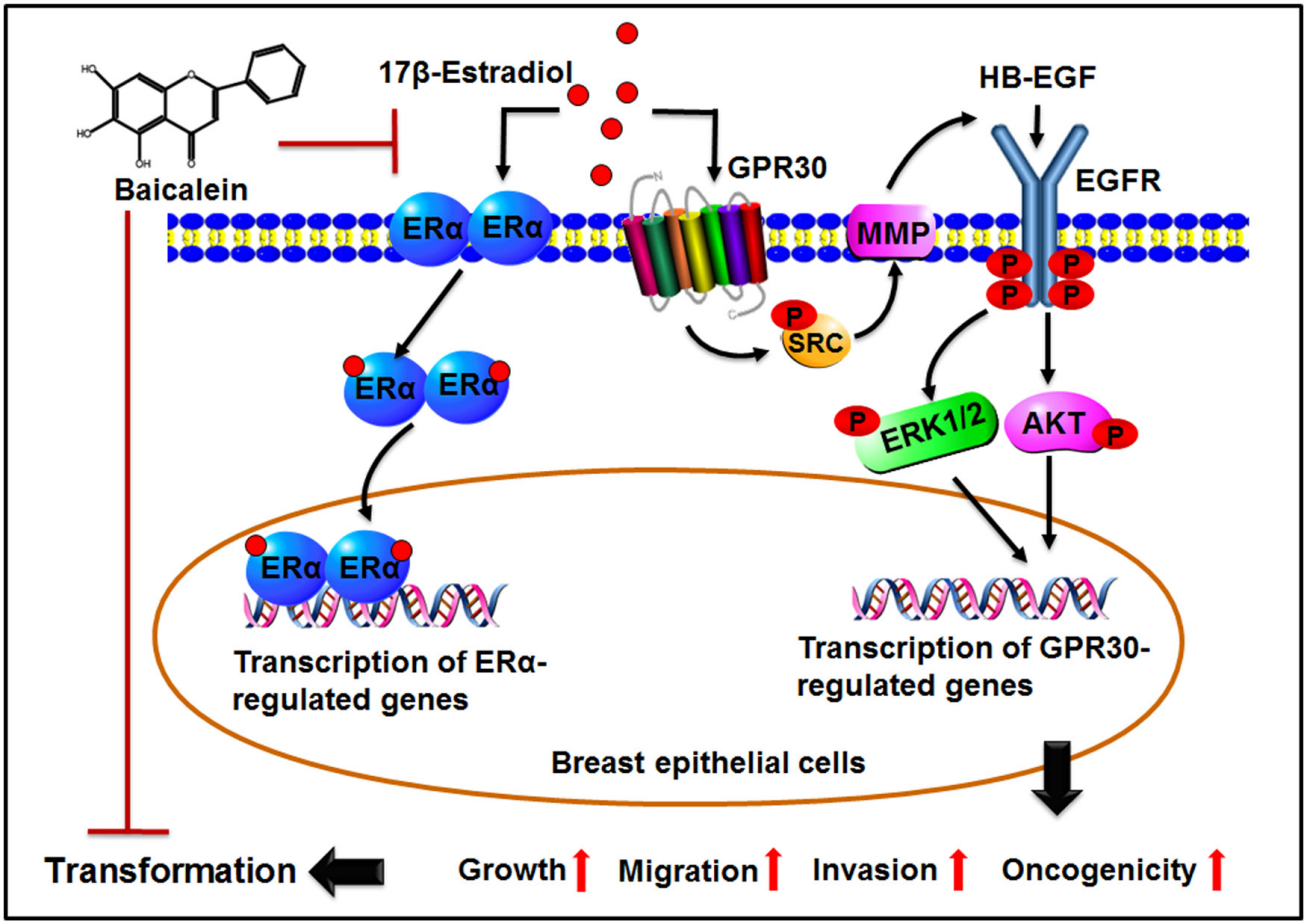
Illustration depicting the possible mechanism underlying the preventive effects of baicalein on the 17β-estradiol-induced transformation of breast epithelial cells

The current results demonstrated that baicalein inhibits E2-induced neoplastic transformation and the cancerous phenotype of the breast epithelium. Moreover, baicalein suppressed the E2-mediated activation of two key regulatory pathways involved in breast carcinogenesis: ERα and GPR30 (Figure 7). Targeting the two pathways simultaneously could represent a novel promising pharmacological approach for the chemoprevention of estrogen-dependent breast cancer that expresses one or both receptors from the onset or following tumor progression. Together, epidemiologic studies, preclinical results, and the positive safety profile encourage the development of baicalein as a potential preventative or therapeutic drug for reducing the risk of breast cancer.

## MATERIALS AND METHODS

### Chemicals

Baicalein (456119) and E2 (E2758) were obtained from Sigma-Aldrich (St. Louis, MO, USA) and dissolved in dimethyl sulfoxide (DMSO) as 0.1 M stock solutions; they were stored at –20°C and 4°C, respectively. Matrigel (356237) and growth factor reduced (GFR) Matrigel (356231) were obtained from BD Biosciences (Bedford, MA, USA). Trypan blue (T6146) was purchased from Sigma-Aldrich. Low melting temperature agarose was purchased from Lonza (50101, Rockland, ME, USA). Paraformldehyde and 4, 6’-diamidino-2-phenylindole dihydrochloride (DAPI, BD5010, working concentration: 1 μg/ml) were purchased from Bio-World (Dublin, OH, USA). The nuclear and cytoplasmic protein extraction kit was purchased from Beyotime Biotechnology (P0027, Nanjing, China). The GPR30 (N-15)-R antibody (sc-48525-R, 1:800) was purchased from Santa Cruz Biotechnology, Inc. (Santa Cruz, CA, USA). Primary antibodies against p-ERK (T202/Y204) (AP0484, 1:1000), ERK1/2 (L352) (BS1112, 1:1000), and GAPDH (1A6) (MB001, 1:1000) were purchased from Bio-World. Primary antibodies for ERα (D8H8) (#8644, 1:1000), p-AKT (Ser473) (193H12) (#4058, 1:1000), AKT (11E7) (#4685, 1:1000), p-EGFR (Tyr1173) (53A5) (#4407, 1:1000), EGFR (D38B1) (#4267, 1:1000), p-SRC Family (Tyr416) (D49G4) (#6943, 1:1000) and SRC (32G6) (#2123, 1:1000) were purchased from Cell Signaling Technology (Beverly, MA, USA). The anti-mouse (sc-2302) or anti-rabbit (sc-2030) IgG horseradish peroxidase-conjugated secondary antibodies (1:3,000) were purchased from Santa Cruz Biotechnology, Inc. The fluorescein isothiocyanate (FITC)-labeled secondary goat anti-rabbit antibody (BS10950, 1:100) was purchased from Bio-World.

### Cell culture and treatment

The MCF-12A and MCF-10A cell lines were purchased from American Type Culture Collection (ATCC, Manassas, VA) and were cultured according to ATCC’s instructions. Both cell lines were cultured in a 1:1 mixture of Ham’s F12 medium and Dulbecco’s Modified Eagle’s medium (DMEM/F-12) (11330032, Gibco, Carlsbad, CA, USA), containing 0.1 μg/mL cholera enterotoxin (C8052, Sigma), 10 μg/mL insulin (I6634, Sigma), 0.5 μg/mL hydrocortisone (H0888, Sigma), 20 ng/mL epidermal growth factor (AF-100-15, PeproTech, London, United Kingdom), and 5% horse serum (HS; 1312396, Gibco). All cell lines were incubated at 5% CO_2_ at 37°C.

Because phenol red (PR) has weakly estrogenic effect [49], both cell lines were treated with 20 nM E2 [4, 50] in the presence or absence of different concentrations of baicalein in PR-free DMEM/F12 (Gibco, 11039021) supplemented with the factors mentioned above. Cells were treated continuously for 5–10 weeks. The cells were passed when the confluence reached 85–90%; they were passed approximately twice a week; and the media were changed every 2 or 3 days. And the followed experiments were performed in PR-free DMEM/F12 added supplementary factors with or without serum.

### Trypan blue dye exclusion assay

Cell growth was assessed using the trypan blue dye exclusion method with manual cell counting with a hemocytometer (Qiujing, Shanghai, China). The cells were collected and plated at a density of 5 × 10^4^ in 1 ml/well in 12-well plates and then incubated for 4 days. The cell numbers were counted every 24 h, and a cell proliferation curve was plotted based on the number of cells counted at different time points.

### Wound healing assay

Cells were seeded into 12-well plates and grown to 90% confluence. The cell monolayers were wounded with a micropipette tip and rinsed with PBS to remove any floating cells. Then, the cells were incubated in serum-free medium for 24 h and images were captured at 0 and 24 h after wound application.

### Invasion assay

The invasive ability of the cells was measured using a transwell chamber assay with 8-μm pore size (R3NA43983, Millipore, Billerica, MA, USA) membranes coated with Matrigel. The cells were trypsinized and suspended at a final concentration of 5 × 10^5^ cells/ml in serum-free medium. The cell suspension was added to the upper chamber, and medium containing 5% HS was added to the bottom chamber as a chemoattractant. After incubation for 24 h, the upper surfaces of the membranes were swabbed to remove non-invaded cells, and any cells that were attached to the lower surface were fixed in 100% methanol, stained with hematoxylin and eosin (H&E) (Beyotime Institute of Biotechnology).

### Soft agar colony formation assay

The 0.6% agarose in medium was solidified onto six-well plates as a bottom layer. The cells (5000 cells/well) were the resuspended in medium containing 0.33% agarose and plated in the plates as the second layer. After solidifying, 1 mL of medium was plated on top of the second agar layer to prevent overdrying. Cells were fed with fresh medium every 3 days and incubated for 21 days. The plates were stained with 0.005% crystal violet and photographed.

### Three-dimensional (3D) cultures on Matrigel

The cells were cultured in Matrigel as reported previously [20]. Briefly, wells in an eight-chamber slide were pre-coated with a layer of GFR Matrigel (60 μl/well) and allowed to polymerize for 30 min. The cells were counted and diluted in assay medium, and 400 μl of cells (5000 cells/well) was plated on top of the solidified Matrigel. The assay medium contained 2% GFR Matrigel, 5 ng/ml EGF, 2% HS, 0.5 μg/ml hydrocortisone, 100 ng/ml cholera toxin, and 10 μg/ml insulin. The cells were incubated for 12 days, and the media were replaced every 4 days with fresh assay medium. Images were captured at 0, 4, 8, and 12 days after seeding using a Leica DMI 1 microscope (Leica, Wetzlar, Germany) at 400 × magnification.

### Immunodeficiency (NOD/SCID) mice models

Investigation has been conducted in accordance with the ethical standards and according to the Declaration of Helsinki and the regulations of the State Food and Drug Administration (SFDA) of China on animal care and use of laboratory animals, and the study protocol was approved by the Committee on the Ethics of Animal Experiments of Guizhou Medical University.

Female non-obese diabetic/severe combined immunodeficiency (NOD/SCID) mice aged 6–9 weeks weighting 20–24 g were purchased from Western Biotechnology Inc (Chongqing, China). The mice were raised in air-conditioned pathogen-free rooms under controlled lighting (12 h light/day) and fed with standard laboratory food and water.

The cells were trypsinized, diluted in PBS, and were mixed with Matrigel at 1:1 ratio. Cell mixtures from different treatment groups (2×10^6^ in 50 μl) were separately implanted into the mammary fat pads of NOD/SCID mice after treatment for 10 weeks (n = 6). All mice received an intramuscular injection with exogenous estrogen (0.3 mg/kg) once 3 days before inoculation with cells, and the same dose of estrogen was provided once per week until the end of the experiment [51, 52]. The tumor volume was calculated every other day using caliper measurements from the time of tumor detection for 30 days. Tumor size was calculated according to the formula:

Tumor size = 0.5 × a × b × b,

where a is the tumor length and b is the tumor width in millimeters.

### Docking study

The structures of ERα and GPR30 were deposited in the protein databank and were used with SWISS-MODEL to build a homology model (ERα PDB code 3ERT, and GPR30 PDB code 1G50). A molecular docking study was performed using the Sybyl-X 1.2 protocol to analyze the receptor-ligand complexes of baicalein and E2 with the ERα and GPR30 LBDs.

### Western blotting

Western blotting was performed as described previously [18]. Digital images of the blots were photographed using a ChemiDoc XRS+ system and analyzed with Image Lab™ Software, Version 5.2 (Bio-Rad, Hercules, CA, USA).

### Immunofluorescence microscopy

Subcellular localization of ERα was analyzed as described previously [53]. The images were captured with a confocal microscope at 1000 × magnification (FV1000; Olympus, Tokyo, Japan).

### Electrophoretic mobility shift assay (EMSA)

Nuclear extracts were prepared and electrophoretic mobility shift assays were conducted according to the manufacturer’s instructions (EMSA kit; GS009, Beyotime, Nanjing, China). Double-stranded probes, including non-biotinylated wild-type ERE, biotinylated wild-type ERE, and non-biotinylated mutant ERE oligonucleotides (ERE-wt 5′-GGA TCT AGG TCA CTG TGA CCC CGG ATC-3′, 5′-GAT CCG GGG TCA CAG TGA CCT AGA TCC-3′; ERE-mut 5′-GGA TCT ACG TAA CTG TTA CGC CGG ATC-3′, 5′-GAT CCG GCG TAA CAG TTA CGT AGA TCC-3′) were custom synthesized by Beyotime. The membrane was visualized using a streptavidin-horseradish peroxidase conjugate coupled to chemiluminescence using a SuperSignal^®^ West Femto Maximum Sensitivity Substrate kit (#34096, ThermoScientific/Pierce). The results were photographed using a ChemiDoc XRS+ system.

### Quantitative real time PCR (RT-PCR)

Total RNA was extracted using an RNA Extraction Kit (Code No. 9767, TaKaRa, Dalian, China), and cDNA was synthesized using a strand complementary DNA synthesis kit (Code No. 6210A, TaKaRa). The mRNA levels were measured using SYBR Green PCR core reagents (Code No. RR037A, TaKaRa) and a CFX Connect™ Real-Time PCR Detection System (Bio-Rad). The primers were synthesized by Sangon Biotech (Shanghai, China) and are listed in Supplementary Table 1. The mRNA levels were quantified using the 2^−ΔΔCt^ method.

### Statistical analysis

Statistically significant differences were calculated using one-way ANOVA followed by the Bonferroni posthoc test for multiple-group comparisons. P < 0.05 was considered to indicate a significant difference and data are expressed as means ± SEM. The data presented were obtained from three independent experiments.

## SUPPLEMENTARY TABLE


