# Supplementary material for: Baicalein has protective effects on the 17β-estradiol-induced transformation of breast epithelial cells
Source: Oncotarget. 2017 Jan 2;8(6):10470–84. doi: 10.18632/oncotarget.14433 (PMC5354673; doi:10.18632/oncotarget.14433)
Supplement: Supplementary file 1 [file oncotarget-08-10470-s001.pdf]

Baicalein has protective effects on the 17β-estradiol-induced transformation of breast epithelial cells

SUPPLEMENTARY TABLE

Supplementary Table 1: The sequence of the primers

gene	Sequence
CYCLIN D1	Sense: 5’-GTCTGTGCATTTCTGGTTGCA-3’
	Antisense: 5’-GCTGGAAACATGCCGGTTA-3’
pS2	Sense: 5’-GCCCCCCGTGAAAGAC-3’
	Antisense: 5’-CGTCGAAACAGCAGCCCTTA-3’
c-FOS	Sense: 5’-CGAGCCCTTTGATGACTTCCT-3’
	Antisense: 5’-GGAGCGGGCTGTCTCAGA-3’
CTGF	Sense: 5’-ACCTGTGGGATGGGCATCT-3’
	Antisense: 5’-CAGGCGGCTCTGCTTCTCTA-3’
EGR1	Sense: 5’-GCCTGCGACATCTGTGGAA-3’
	Antisense: 5’-CGCAAGTGGATCTTGGTATGC-3’
CRY61	Sense: 5’-ACTTCATGGTCCCAGTGCTC-3’
	Antisense: 5’-AAATCCGGGTTTCTTTCACA-3’
GAPDH	Sense: 5’-GAAGGTGAAGGTCGGAGTCA-3’
	Antisense: 5’-GAAGATGGTGATGGGATTTC-3’
